# Saving babies’ lives project impact and results evaluation (SPiRE): a mixed methodology study

**DOI:** 10.1186/s12884-018-1672-x

**Published:** 2018-01-30

**Authors:** Kate Widdows, Holly E. Reid, Stephen A. Roberts, Elizabeth M. Camacho, Alexander E. P. Heazell

**Affiliations:** 1 Maternal and Fetal Health Research Centre, School of Medical Sciences, University of Manchester, St Mary’s Hospital, Oxford Road, Manchester, UK; 2 Centre for Biostatistics, Institute of Population Health, Manchester Academic Health Science Centre, University of Manchester, Manchester, UK; 3 Manchester Centre for Health Economics, Division of Population Health, Health Services Research, and Primary Care, University of Manchester, Oxford Road, Manchester, M13 9PL UK; 4 Manchester Academic Health Science Centre, St. Mary’s Hospital, Central Manchester University Hospitals NHS Foundation Trust, M13 9WL, Manchester, UK

**Keywords:** Stillbirth, Perinatal mortality, Smoking cessation, Fetal growth restriction, Reduced fetal movements, Fetal monitoring, Intrapartum fetal monitoring, Quality improvement

## Abstract

**Background:**

Reducing stillbirth and early neonatal death is a national priority in the UK. Current evidence indicates this is potentially achievable through application of four key interventions within routine maternity care delivered as the National Health Service (NHS) England’s Saving Babies’ Lives care bundle. However, there is significant variation in the degree of implementation of the care bundle between and within maternity units and the effectiveness in reducing stillbirth and improving service delivery has not yet been evaluated. This study aims to evaluate the impact of implementing the care bundle on UK maternity services and perinatal outcomes.

**Methods:**

The Saving Babies’ Lives Project Impact and Results Evaluation (SPiRE) study is a multicentre evaluation of maternity care delivered through the Saving Babies’ Lives care bundle using both quantitative and qualitative methodologies. The study will be conducted in twenty NHS Hospital Trusts and will include approximately 100,000 births. It involves participation by both service users and care providers. To determine the impact of the care bundle on pregnancy outcomes, birth data and other clinical measures will be extracted from maternity databases and case-note audit from before and after implementation. Additionally, this study will employ questionnaires with organisational leads and review clinical guidelines to assess how resources, leadership and governance may affect implementation in diverse hospital settings. The cost of implementing the care bundle, and the cost per stillbirth avoided, will also be estimated as part of a health economic analysis. The views and experiences of service users and service providers towards maternity care in relation to the care bundle will be also be sought using questionnaires.

**Discussion:**

This protocol describes a pragmatic study design which is necessarily limited by the availability of data and limitations of timescales and funding. In particular there was no opportunity to prospectively gather pre-intervention data or design a phased implementation such as a stepped-wedge study. Nevertheless this study will provide useful practice-based evidence which will advance knowledge about the processes that underpin successful implementation of the care bundle so that it can be further developed and refined.

**Trial registration:**

www.clinicaltrials.gov NCT03231007 (26th July 2017)

## Background

Maternity services in England aim to deliver high quality care and outcomes for both mother and baby are improving [1]. The stillbirth rate and neonatal mortality rate fell by 12% and 10% respectively between 2010 and 2015 [2]. However, comparison to other high income countries suggests that more can still be done to reduce stillbirth rates in the UK [3]. One in every 260 babies born in the UK are stillborn each year [1]. In 2015, the UK ranked 24th out of 49 high income countries and the annual rate of reduction of 1.4% is significantly lower than comparable countries (e.g. 6.8% in the Netherlands) with about a 33% variation in rates between regions [3, 4]. The recent Lancet Ending Preventable Stillbirth Series called for efforts to address the disparity in stillbirth rates between, as well as within, individual countries [3].

A recent Confidential Enquiry of normally-formed term antepartum stillbirths revealed that almost half are potentially avoidable if improvements were made in at least one element of antenatal care [5]. To address this, in November 2015 the UK government launched a national Maternity Transformation Programme (MTP) to promote safer care for women and newborns with the ambition of reducing stillbirths and early neonatal deaths by 20% by 2020 and by 50% by 2030 [6]. As part of the MTP, in April 2015 the National Health Service (NHS) in England launched the Saving Babies’ Lives (SBL) care bundle specifically designed to reduce stillbirths and early neonatal deaths [7]. It is a programme aimed at improving outcome by improving the quality of care during pregnancy and outcomes through four key elements, established using the best current practice/evidence in reducing stillbirth and early neonatal death by: 1) Reducing cigarette smoking in pregnancy, 2) Improving detection and management of fetal growth restriction (FGR), 3) Improving awareness and management of reduced fetal movement (RFM) and 4) Promoting effective fetal monitoring during labour.

These four elements are predicted to improve the identification of babies at greatest risk with particular application to term antepartum stillbirths that are potentially avoidable. It encompasses two key recommendations from the 2015 Confidential Enquiry (monitoring fetal growth [8] and managing reduced fetal movements [5]) as well as key public health action to reduce smoking in pregnancy and the importance of effective fetal monitoring during labour [9–11].

It is imperative that public health interventions to reduce stillbirths are based upon robust evidence. Although the elements were derived from national clinical guidelines and widely accepted best practice [12–15], more primary data is needed to assess the effectiveness of the care bundle at reducing stillbirth rates. The implementation phase of the care bundle provides a unique opportunity to collect this evidence. The process of implementation (e.g. the interventions) must also be evaluated to determine what qualities underpin successful implementation and the needs of different maternity units in doing so. In this way we can, subject to the limitations of the available data and the observational nature of the study, determine to what extent the care bundle provides clinically effective interventions.

To determine how contemporary practice already aligns with the care bundle and how maternity units are working towards implementation, a survey was sent by NHS England to all maternity units across the UK in 2015. Of the 99 units that responded, all were implementing at least one element of the care bundle and there was significant variation in the degree of implementation of the different elements both between and within units. Forty-nine of these volunteered to be Early Implementer sites. The current study will focus on the Early Implementer sites who have been piloting the care bundle since its launch.

Reasons for the national variation in implementation are likely to be centred on resource, structural and institutional influences. This study is designed to better understand these factors and will focus on a small number of maternity units as part of a detailed evaluation rather than a superficial evaluation of a larger number of hospitals. Twenty maternity units were selected as study sites from the Early Implementer’s according to Rogers Model of Innovation [16]. The *diffusion of innovation* theory seeks to explain how innovations are taken up in a population and provides valuable insight into the process of change. Study sites were categorised as innovators, early adopters, late adopters and low adopters according to their level of implementation for the care bundle (from the 2015 survey). The units vary in size (in terms of number of births per year) and a range of sites from across different strategic clinical networks are included to ensure that data is geographically representative. Based on the number of study sites involved, this provides us with an estimated 100,000 births per year. This equates to 13% of the total annual births in the UK (779,688 livebirths in UK) and will potentially include approximately 390 stillbirths per year assuming a stillbirth rate of 3.9 per 1000 total births (2015) [1].

## Methods

### Study aims and objectives

The overall purpose of the study is to determine the impact of the care bundle on UK maternity services and perinatal outcomes. It specifically aims to: 1) Evaluate the effectiveness of the care bundle in reducing stillbirth, 2) Assess the degree to which each specific element has been implemented, 3) Understand the processes and contexts of implementation success and 4) Estimate the cost of implementing the care bundle. To achieve these aims, the study has a number of objectives. The primary objective is to compare stillbirth rates before and after implementation of the care bundle. Secondary objectives include: i) to explore factors associated with reduction in stillbirth and post-implementation rates, ii) to assess the impact of the care bundle on other relevant clinical endpoints, iii) to assess implementation stages for each element and change from pre-implementation, iv) to explore leadership, governance and workforce culture with implementation success, v) to explore patient and staff views of antenatal care in relation to the care bundle, vi) To estimate the resources required to implement the care bundle and vii) To estimate the cost of implementing the care bundle per stillbirth avoided post-implementation.

### Study design

This study is a multicentre mixed-methods evaluation to determine the impact of the care bundle on maternity services and perinatal outcomes. The evaluation will last for a period of 13 months commencing 1st March 2017 until 31st March 2017. Data collection will begin in July 2017 and end in December 2017. Data analysis, report writing and dissemination of research findings are anticipated to take place between January 2018 and March 2018.

### Study setting

The study will be conducted in twenty NHS Hospital Trusts from eight NHS Strategic Clinical Networks. These include the South West, Thames Valley, North West Coast (formerly Lancashire and South Cumbria, and Cheshire and Merseyside), North East, Yorkshire and Humber, Greater Manchester and East Cheshire, and the East Midlands SCNs. Where there are multiple hospitals per NHS Trust, study sites will be asked to include data from all relevant hospitals. The Trust will be the unit of analysis (centre) for implementation measures. However, if on review substantive differences are identified between sites within Trusts (e.g. restructured Trusts) sites may be split prior to evaluation of the outcome data.

All participating Trusts are currently implementing the Saving Babies’ Lives care bundle; however, there is variation in terms of degree of implementation across Trusts. This sampling strategy aims to allow assessment of implementation success across a range of settings and the processes and contexts of implementation. Their active participation in the study (including extraction of historic data) is anticipated to last for a period of 6–9 months.

As there was no opportunity to be involved in the implementation process, or access the services prior to the implementation, we have pragmatically adopted a pre-post study design using routinely collected data, augmented by cross-sectional targeted data collection in the post-implementation period. This design will provide evidence of the degree to which stillbirth rates and associated clinical and process outcomes have improved following implementation of the care bundle, accepting that causal attribution to the care bundle (as a whole or any element of the bundle) will be provisional. We have adopted an extended pre-implementation observation period to allow the discernment of time trends external to the bundle, and a range of implementation levels and times to allow some attribution to care bundle elements.

As with any quality improvement, the measurement of key outcomes is critical and we have taken a pragmatic approach to assessing the various outcomes and processes for each intervention. The outcome measures being evaluated in this study were informed by a small feasibility study (conducted from May 2016 to January 2017) involving some of the innovator sites. This determined the availability and reliability of collecting data for each of the care bundle interventions.

### Plan of investigation

**Fig. 1:**
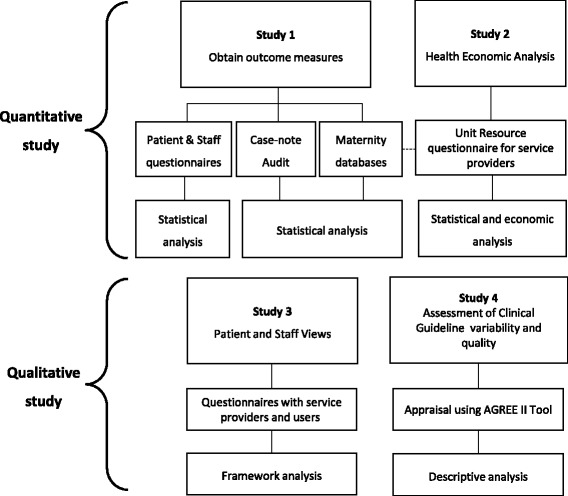
Plan of Investigation identifying quantitative and qualitative components

The evaluation methodology is broadly categorised into two work streams; 1) quantitative studies and 2) qualitative studies. The overall plan of investigation is summarised in relation to the study objectives (Fig. 1). This mixed-methods approach will allow description of the intervention characteristics, the setting, the characteristics of the individuals involved and the processes and impact of implementation.

The first two objectives (referred to as Study 1 in Fig. 1) will utilise routinely collected electronic data which will be extracted from maternity databases alongside targeted case-note audits of selected groups of patients. In addition, questionnaires with patients and healthcare professionals will be employed to obtain a small number of outcome measures. Implementation data will be obtained from the sites’ Clinical Directors or Head of Midwifery using a Unit Resource and Leadership questionnaire. This data will be analysed to estimate changes in stillbirth rates, the degree to which each element has been implemented and to explore associations between clinical outcomes and implementation.

The fourth objective will be addressed by outcome and process measures collected from the organisational lead questionnaires and routinely collected data from Study 1 to estimate the cost of implementing the care bundle and cost per stillbirth avoided. The relationship between the degree of implementation and extent of resources used will also be explored. The third objective will be addressed by open-ended questions administered as part of the questionnaires for patients and healthcare professionals to learn more about views and experiences of maternity care in relation to the care bundle.

Finally, clinical guidelines will be appraised using the AGREE II Tool [17] to explore the variability and quality of guidelines between Trusts and the clinical credibility and reliability of guideline recommendations.

### Data collection

Outcome measures (Table 1) will be collected from three primary sources; Maternity Information systems (MIS), the GROW-App™, (a subsidiary online application for the generation of the Gestation Related Optimal Weight (GROW) curve [18]), and targeted case note audits on selected subgroups of patients. A small number of outcomes will be collected from the patient questionnaire where they are not available in any routine records.

**Table 1:** Clinical Outcome Measures and their respective definitions

Clinical Outcomes	Measurement	Definition
Primary Outcome	Stillbirth Rate	The death of a baby before or during birth after 24 weeks of gestation [24] expressed as a proportion of live births.
Secondary Outcomes	Term, normally formed singleton stillbirths.	Stillborn infants with no evidence of congenital anomaly
	Preterm birth rate	Births before 37 weeks
	Gestation at birth	
	Induction of labour rate	Proportion of women with induction of labour (excluding women who have elective CS)
	Rate of normal vaginal delivery	Proportion of all births by normal vaginal delivery
	Rate of instrumental vaginal delivery	Proportion of all births by instrumental vaginal delivery
	Rate of Caesarean section	Proportion of babies born by Caesarean section (emergency + elective procedures)
	Rate of Emergency Caesarean Section	Proportion of births by emergency Caesarean section
	Birthweight	
	Birthweight centile	Calculated using GROW-Centile software
	Rate of admission to neonatal intensive care unit (NICU)	Proportion of babies admitted to NICU for any indication after delivery
	Number of babies therapeutically cooled	Proportion of babies receiving therapeutic cooling for hypoxic ischaemic encephalopathy
	Number of reported clinical incidents	Number of clinical incidents (i.e. clinical events (massive obstetric haemorrhage) as opposed to non-clinical e.g. staff shortage) reported on hospitals incident reporting system.

Data will be extracted retrospectively from electronic Maternity Informatics Systems (MIS) at each site. Data will be collected for all singletons and multiple births per hospital. Trusts will be asked to provide approximately 5 years of outcome data. This will comprise 3 years of baseline data pre-launch of the care bundle and at least 2 years of outcomes post-launch. Sites will be requested to submit data on two occasions. Once at the start of the study period for all pre-post launch data up until the study start date e.g. April 2017 (Dataset 1). Sites will then be required to submit data 2 years after the launch for a refresh of all post-launch data (Dataset 2). A Data Collection Proforma (DCP) (Microsoft Excel) will be provided to all sites to facilitate collection of data by a delegated team member at each trust.

A small number of outcomes relating to the antenatal detection of SGA babies will be collected for sites that are enrolled in the Perinatal Institute’s Growth Assessment Protocol (GAP) from data held in the GROW-App [18]. Researchers at the sponsor site will co-ordinate directly with the PI to retrieve this data (monthly figures). These data will be collected from the post-launch period only.

Case-note audit of patients’ antenatal records will be carried out to assess a small number of process outcomes from the care bundle interventions. Study sites will be required to carry out three separate case-note audits on different populations of patients as follows: general births, SGA pregnancies and RFM pregnancies. These are based on 20 or 40 consecutive patients meeting the criteria in two specified collection periods. SGA pregnancies will either be identified via the GAP programme or from data submitted to the study team. Women attending with RFM will be identified from triage or antenatal assessment unit activity logs. In addition, it may be necessary for hospitals to review stillbirth cases should this information be unavailable from their MIS. Data will be provided as fully anonymised individual case records. Study sites will be provided with Audit Proformas to facilitate this process and will have 6 months (April until September 2017) to submit the data.

NHS Trusts using GAP-SCORE (Standardised Case Outcome Review and Evaluation) for the review of clinical care in the ‘missed case’ of fetal growth restriction will be encouraged to use the online audit tool to assess the Care Bundle’s “*Algorithm and Risk Assessment Tool for Screening and Surveillance of fetal growth in singleton pregnancies.*” The online audit should be carried out on the same 20 SGA singleton pregnancies (post-implementation) reviewed as part of the SGA case-note audit outlined above; this includes both missed and known SGA cases. NHS Trusts that are currently enrolled in GAP but are not yet using GAP SCORE are encouraged to perform the online audit but this is entirely optional. Data will be provided as fully anonymised individual case records. Individual Trusts will not be identified in any reports.

### Qualitative study

Questionnaires will be used to explore the views of patients and healthcare professionals towards maternity care in relation to the care bundle including implementation practices. In addition, a small number of outcomes that are not routinely available in maternity databases will be collected using questionnaires. The questionnaire includes both close-ended and open-ended questions and should take no more than 15 min to complete. Participants will be asked to provide basic demographic information once they have completed the questionnaire. Questionnaires are intended to be carried out at all relevant sites within the Trust where possible.

### Service-user questionnaire

Women will be eligible to complete the questionnaire if they received their antenatal care, delivered and were discharged from the same maternity unit, and if they have given birth after 28 weeks of gestation. Women who are ≤16 years of age, women who cannot understand/not fluent in English (to enable consent without interpreter), with multiple pregnancy, fetuses known to have any congenital or severe structural abnormalities, and/or home births will be excluded from this part of the study.

Eligible women on the postnatal ward will be identified and approached by their midwife or doctor to ask if they would like to complete the questionnaire. Patients will be given information about the questionnaire and reassured that their responses will be anonymous. A proportionate approach to consent will be taken, i.e. by completing the questionnaire the respondent has consented to participating. Women will be reassured that the care they and their babies receive will not be affected by completing the questionnaire. All participants will be informed that completing the questionnaire is voluntary and will be explicitly informed prior to completing the questionnaire that once they submit their responses they will not be able to withdraw from the study (to ensure anonymity). Participants will have the option to be entered into a £100 prize draw for an Amazon voucher as a gesture of thanks for their participation, and will be given the option to provide their contact details in a separate document that will not be linked to the questionnaire responses to maintain anonymity.

For hospitals with Wi-Fi access on the postnatal ward, participants will be given an electronic tablet to complete the questionnaires online. Data will be entered into Select Survey.NET which is the University of Manchester’s secure online survey tool. Electronic tablets will be provided for hospital sites where unavailable to facilitate online completion. Responses will be available immediately to the research team once participants submit the questionnaire online. Alternatively, questionnaires will be completed using pen and paper where study sites do not have Wi-Fi access. It will be the responsibility of the site Principal Investigator (PI) to collect and securely store completed paper questionnaires prior to sending to the sponsor site.

For each site, all patients delivering in the unit in a designated 2 week period (during the study period) should be asked to complete the questionnaire prior to discharge. Smaller units who deliver less than 50 babies per week will be asked to collect for 3 weeks to reduce the bias towards larger centres. It is not practical to translate the questionnaire into the large number of potential languages that may be required to accommodate all non-English speakers. As translation services will be required to complete discharge procedures, centres will be asked to ensure that these services are available to aid questionnaire completion. The response rate across ethnic groups will be monitored to determine the extent of any bias due to non-provision of appropriate translation facilities.

### Healthcare professional questionnaire

Health professionals will be asked to complete a questionnaire, this includes midwives (antenatal ward, antenatal clinic, community-based staff, antenatal assessment unit and labour ward), sonographers, junior doctors and consultant obstetricians, Clinical Directors and Heads of Midwifery. Staff must have been employed in their current Trust prior to the launch of the care bundle initiative in April 2015 for them to be eligible to complete the questionnaire. Staff members will not be eligible to complete the questionnaire if they were employed after the care bundle was implemented.

Eligible healthcare professionals will be identified and recruited by members of the site research team. The online version (Select Survey.NET) of the questionnaire is intended be sent to all clinical staff who deliver maternity care as part of the care bundle. It will be the responsibility of the PI at each hospital site to distribute the link to all eligible healthcare professionals. The sample size for the Health Professionals questionnaire is determined by the unit size and number of eligible participants within each discipline. It is intended to survey all eligible professionals. A proportionate approach to consent will be taken, i.e. by completing the questionnaire the respondent has consented to participating. Participants will have the option to be entered into a £100 prize draw for an Amazon voucher as a gesture of thanks for their participation. The response rate for each site will be monitored and reported. It will be distributed to all eligible clinical staff within a three month period of the start of the study.

### Unit resource and leadership questionnaire

A Unit Resource and Leadership questionnaire will be used in this study to capture the structural and institutional characteristics of each maternity unit and resource usage for the health economic analysis. Organisational leads (e.g. Clinical Directors and Heads of Midwifery) at each hospital will be asked to complete the online questionnaire using the Select Survey Tool. This questionnaire contains a combination of closed-ended and open-ended questions. It will be the responsibility of the PI at the sponsor site to distribute the link to the online questionnaire to the Clinical Director or Head of Midwifery. One Unit Resource and Leadership questionnaire is expected to be returned per hospital. The questionnaire should be completed by the end of July 2017.

### Guideline appraisal

Study sites will be required to provide their antenatal and intrapartum care guidelines for quality appraisal. Guidelines will be independently scored by two members of the sponsor research team using the accredited Appraisal of Guidelines for REsearch and Evaluation tool (AGREE II) [17]. Study sites will be asked to provide guidelines as soon as enrolment is complete. Study sites will receive a breakdown of their guideline scores and potential recommendations for improvements following study completion. Data will be compared to a prior study of guidelines for element 3 [19]. The sponsor will not share guidelines scores with other study sites without prior approval.

### Statistical analysis

The sample sizes for the various datasets are largely determined pragmatically by the constraints of time and the need to have a reasonable pre and post launch period to assess trends, with an inevitably staggered true implementation. Based on a prevalence of 4.7 normally formed singleton stillbirths per 1000 total births in 2014 [1], the potential annual number of stillbirths detected in this study will be 470 per 100,000 total births estimated across all study sites. Around 30% of these would be expected to be normally–formed term singletons. Conservatively, (assuming we obtain 2/3 of the planned dataset) a two year pre versus one year post-comparison would be estimated to have 80% power to detect a drop in the primary stillbirth rate from 4.7 to 3.9/1000 – a 17% reduction. For normally formed term singletons we would expect to be able to detect a reduction in rate from 1.4 to 1.0/1000 –a 40% reduction in the subgroup where the bundle is expected to be most effective.

The audit sample sizes are heavily constrained by feasibility. The targeted audits of specific interventions for RFM (post) and SGA (pre and post) with 20 per audit per site gives a total sample size of 400. This is sufficient to give overall estimates of compliance rates of ±5% on rates of ~ 50% and ±3% on rates ~ 90%. The sample size (with 40 events for a 10% non-compliance rate) should allow some limited investigation of associations with implementation factors. The general audit (post only) has a doubled sample size reflecting the greater number of these patients and allowing somewhat more scope for exploration.

The patient questionnaire sample size is determined by the need to focus on a fixed period of time to allow computation of completion rate, the need to rotate a limited number of data collection tools between sites (e.g. electronic tablets) and to encourage site compliance with a short focussed collection period. The primary consideration is the number in the at risk subgroups who smoke at booking or present with RFM. A collection period of 2 weeks should yield a total sample size of approximately 2300 with a 60% completion rate. Across all study sites this will give 140–345 RFM pregnancies (based on an incidence of 6–15% of women presenting with RFM [20, 21] and 240 pregnancies in smokers (based on a prevalence of 10.6% of women smoking at the time of delivery [22]. These numbers again allow reasonable precision (e.g. ± 6% on prevalence of approximately 30% in 240 smokers) albeit with little scope for exploration of association with implementation or case-mix factors.

The sample size for the Health Professionals Views questionnaire is determined by the unit size and number of eligible participants within each discipline. It is intended to survey all eligible professionals. The response rate will be monitored and reported.

### Data analysis

All outcomes will be summarised using appropriate descriptive statistics. Data summaries will be presented prior to the care bundle launch and for the post-launch period (from 6 months after the launch until the end of data collection). Compliance with each element is assessed by a number of process measures (Table 2). This is a dynamic process and it is not certain at the outset which summary measures will be appropriate to quantify compliance and allow meaningful comparisons of patient outcomes with compliance. Therefore, the research team will review the entirety of the data set on compliance, visualising it graphically, without considering patient outcomes and anonymised to unit. The research team will review this data and select appropriate measures prior to any sight of outcome data. If any substantive differences are observed within any Trusts, consideration will be given to splitting that Trust into multiple sites for analysis.

**Table 2:** Planned process measures relating to each element of Saving Babies Lives Care Bundle

Element	Outcome Type	Process Outcome
Element 1 Smoking and Pregnancy	Primary Outcome	Proportion of women smoking at delivery
	Secondary Outcomes	Proportion of women offered carbon monoxide (CO) test
		Proportion of women accepting CO test
		Proportion of women referred to smoking cessation
		Proportion of women ceasing smoking between booking and delivery
		Proportion of women referred to smoking cessation with a positive CO test
Element 2: Detection of SGA	Primary Outcome	Proportion of SGA singletons detected prior to delivery
	Secondary Outcomes	Proportion of all singletons with growth charts in notes
		Proportion of SGA pregnancies with estimated fetal weight (EFW) plotted on growth chart
		Proportion of SGA pregnancies with symphysis fundal height (SFH) plotted on growth chart
		Proportion of SGA pregnancies with EFW correctly plotted on growth chart
	Exploratory Outcomes	Proportion of babies identified as SGA during pregnancy that were appropriate for gestation age (AGA) at birth (false positives)
		Proportion of babies identified as AGA during pregnancy that were SGA at birth (false negatives)
		Proportion of pregnancies with customised growth chart
		Birthweight centile at last scan (by EFW measurement)
		Number of third trimester growth scans
Element 3: Patient information provision and RFM management	Co-primary outcomes	Proportion of women receiving RFM leaflet
		Proportion of women with RFM managed according to checklist
	Secondary outcomes	Number of women attending antenatal triage clinic
		Proportion of triage women presenting with RFM on at least one occasion
		Proportion of women with RFM who had scan
	Exploratory outcomes	Proportion of women with RFM who had FH monitoring
		Gestation of baby at RFM episodes
		Number of growth scans due to RFM
		Time to scan from reporting RFM
		Number of RFM episodes per pregnancy
Element 4: Effective fetal monitoring during labour	Primary outcome	Proportion of deliveries where both buddy and stickers used
	Secondary outcomes	Proportion of staff completing annual CTG training
		Proportion of pregnancies where escalation protocol was used

Stillbirth and early neonatal death rates submitted by hospital sites to the University of Manchester will be cross-checked against other national sources of data to ensure that all cases have been reported. Incidence data will be checked against information that is routinely collected from the Office of National Statistics, MBRRACE and Each Baby Counts (EBC). Individual case reviews will not be carried out in this study; patient identifiable data will therefore not be required.

Prior to analysis, data will be checked for outliers and invalid values and appropriate decision rules for inclusion/exclusion/correction agreed within the study team and documented. Rates of discarded data will be summarised by pre/post period.

### Planned statistical analysis

Pre-Post Analysis: The primary outcome and other study outcomes captured as monthly counts will be formally analysed using a pre-post analysis by means of longitudinal logistic regression models with a centre random effect. Monthly proportions before the launch (April 2012–April 2015) will be compared with 6 month post-launch to the end of the study period (Oct 2015-Oct 2017). Covariate adjustment for calendar month and case-mix tariff and smoking rates at booking will be carried out.

Interrupted Time Series Analysis: The pre-post analysis will be extended using interrupted time series (ITS) analysis. This will explicitly test for a “step change” following implementation in the possible presence of other time trends.

Exploratory Analyses: The ITS model will be extended to test for changes associated with implementation times of each component at each study site (“unit-specific”). The pre-post model will be expanded to assess whether any changes in the outcomes arising from the implementation of the care bundle are associated with degree of compliance at the final audit date.

Mean data will be analysed using analogous ordinary regression models. Some outcomes may be better modelled as event rates with Poisson regression models and offset for the population size. Either General Estimating Equation (GEE) or Generalised Linear Mixed Model (GLMM) approaches will be used to fit the models depending on computational considerations.

Pre-post Analysis – Audit data: A similar approach will be used to evaluate outcomes available only at two time points, albeit with more restricted longitudinal models.

Post Launch Audit/Questionnaires*:* Single time point post-launch data will primarily be reported descriptively. Where appropriate sample/response weighting will be used to produce questionnaire-derived estimates relative to the sampled population. Exploratory analyses will assess the effect of the degree of implementation of the care bundle and organisational factors on the outcomes, adjusting as far as feasible for case-mix covariates.

### Qualitative analysis

The responses to the open-ended questions from the patient and health care professional questionnaires will be analysed using Framework Analysis, which focusses on identifying and giving meaning to patterns within the data set [23]. Responses will be submitted to inductive line-by-line coding to identify relevance to the research aims from as many different perspectives as possible. The first few responses will be coded independently by different members of the research team. After this, codes will be grouped into categories which will form the analytical framework. Subsequent responses will be indexed using the analytical framework. Data will then be charted into a framework matrix by case (participant) and category. Through interpretation of the matrix, theoretical concepts describing and explaining the similarities and differences in the data will be developed.

### Health economic analysis

The quality and completeness of data will be initially reviewed prior to consideration of appropriate approach for the economic analysis. The economic analysis will explore and present descriptive statistics for key drivers of cost and the resources (staff time, training, equipment) reported by each study site to implement the care bundle (overall and for each element). The costs will be presented from the perspective of the NHS and standardised to a single financial year (2015–16 is anticipated to be the most recent unit costs available at the time of the analysis). National birth data published annually by the Office for National Statistics (ONS) will be used to estimate the nationwide cost of implementing the care bundle (overall and for each element). Data on resource use will include information collected on pregnancy outcome and process outcomes e.g. number of scans (Table 3).

**Table 3:** Components of planned economic and resource evaluation

Resource(s)	Relevant example
Equipment required	Carbon monoxide testing kits
Staff time required	To conduct additional scans
Number of patients attending antenatal triage	
Number of patients presenting with RFM on at least one occasion	
Birth by Emergency Caesarean	
Induction of labour	
Number of third trimester growth scans	
Number of growth scans due to RFM	
Number of antenatal CTGs due to RFM	
Length of stay in NICU	
Length of stay in hospital	

The cost of implementing the care bundle in study sites will be presented alongside the number of stillbirths potentially avoided as per results of the care bundle (estimated from statistical analyses described above). A formal cost-effectiveness analysis will not be conducted due to the limitations of the study design. Uncertainty around the overall cost estimate will be explored using sensitivity analyses to test the impact of key assumptions.

For each site the cost to implement the care bundle for a set number of births (e.g. 1000) will be calculated (as a pragmatic way to account for unit size). The correlation between level of compliance and implementation costs will be explored. The mean costs will be compared between high and low compliant sites using a t-test. Differences between the resources required by different sites to achieve different levels of compliance will be explored in terms of potential trade-offs between input (resources) and output (level of compliance). The impact of case mix tariff and size of unit will be considered depending on the characteristics of available data.

### Data handling and storage

Anonymous data retrieved from MIS and case-note audits will be transcribed into the DCP by the delegated site member. All proformas will be in Microsoft Excel format. We will receive the spreadsheets after extraction is complete at the study site. For ease of data entry and to minimise data entry errors, the database used to collect data in the study will consist of text boxes, dropdown lists and checkboxes to minimise the use of free-text. Individual data items will be validated against a set of rules, for example gestational age can only be entered in days and not weeks and within a specified range. The spreadsheets will include a combination of individual and aggregated (monthly) patient datasets. No identifiable data will be included in these spreadsheets. Files will be saved as .xls using the existing study site names and password protected.

Data from the online questionnaire will be exported from SelectSurvey.NET into an Excel workbook. This will be conducted by the research team at the sponsor site after the submission of all questionnaires. Questionnaires will be assigned a specific study number for analysis purposes. Names and contact details from participants who wish to be entered into the prize draw will be stored separately to the questionnaire responses. This information will be kept strictly confidential and all participant names and contact details will be permanently deleted after 6 months following completion of the study.

All data files will be returned electronically to the sponsor research team. A designated study email address has been established by the sponsor site to permit the secure transfer of data between study sites and the University. Data will be encrypted/password protected for transfer with the password details sent separately. Alternative secure file transfer arrangements are available and all data transfer will conform to University of Manchester guidelines. Any email attachments containing the submitted data will be permanently deleted following data transfer.

All data generated by the study will be preserved and stored with no identifiable details for 5 years after completion of the study, except email addresses collected to notify participants of the publication of findings which will be destroyed once they are no longer needed (i.e. once participants have been notified of publication). Dr. Alexander Heazell will be custodian of the data on behalf of the sponsor. Electronic data will be stored in appropriate data storage devices. All electronic and hard-copy data files will be stored securely in accordance with local policy at University of Manchester. Electronic data will be stored on a secure University of Manchester server that complies with University data security policies or (temporarily) on University owned and fully encrypted laptops. Study data and material may be looked at by individuals from the University of Manchester, from regulatory authorities or from NHS Trusts, for monitoring and auditing purposes and this may well include access to personal information.

### Quality assurance and control

Study sites will be required to identify a Principal Investigator (PI) at each site who will manage local data collection and communicate directly with the study co-ordinator regarding all aspects of the study. Site PIs will be asked to sign all necessary agreements relating to the collection and storage of data. Key members of the site research team will be required to undergo training prior to commencing the study regarding study design, identifying eligible patients, and the collection of data and record keeping. Participating sites will be required to notify the study co-ordinator of any changes in the site research team. Each site team and site Research and Development department will receive the local information pack and exchange agreements (i.e. the Statement of Activities) prior to commencing recruitment at the site.

### Data monitoring

On-site monitoring will be carried out as required, for example due to poor completion of the data collection templates, poor data quality, or low participant recruitment. Sites will be given advance notification of an on-site visit if required and the sponsor site will give written confirmation of the proposed date. All staff involved in the study sites will be given access to source data under these circumstances to facilitate review. The study co-ordinator will be in regular contact with study sites to assess progress with the data collection and address any potential queries. Sites will be asked for missing data and clarification for incomplete or inconsistencies in data where applicable.

This study will be carried out at multiple sites across NHS England Regions. The sponsor is the University of Manchester. The sponsor study site is St Mary’s Hospital, Central Manchester Foundation Trust. This study site has a Chief Investigator - Prof Alexander Heazell and four co-investigators (Dr Kate Widdows; Dr. Elizabeth Camacho, Dr. Steve Roberts and Holly Reid). Dr. Kate Widdows will be the Study Co-ordinator. They will oversee the day-to-day management of the study, including monitoring protocol compliance, safeguarding participants and ensuring quality of data collection. Any problems or queries will be passed to the study co-ordinator at the sponsor site in the first instance. In her absence one of the co-investigators will deputise.

The Study Stakeholder Committee (SSC) will consist of various representatives from third-sector and professional stakeholders with relevant expertise in stillbirth research. The SSC will provide independent oversight of the study and will meet as required and at least twice during the duration of the study. Meetings will be conducted either face-to-face or via teleconference. The SSC will review the data collected on stillbirths and neonatal deaths before discharge and act in accordance with the SSC terms of reference (TOR). The end of the study is planned to be January 2018. The sponsor will notify the research ethics committee that the study has ended and a final study report will be provided within 12 months of the end date.

Health Research Authority (HRA) was obtained in June 2017, which included a review by a NHS Research Ethics Committee (17/WM/0197). Study sites will not be permitted to recruit participants to the study until the PI at the sponsor site has received written confirmation of HRA approval. This study will be performed in accordance with the Research Governance Framework for Health and Social Care and the Data Protection Act 1998.

There is a possibility that service users may find the questionnaire upsetting if they have had a particularly negative pregnancy or birthing experience, although the questionnaire has not been designed to evoke upsetting or sensitive responses. However, some participants who choose to express their views anonymously via the questionnaire may find this beneficial.

### Confidentiality and data protection

Personal data recorded on all documents will be regarded as strictly confidential and will be managed and stored in accordance with the Data Protection Act 1998.The sponsor site will maintain the confidentiality of all study participants’ contact details and will not disclose information by which participants may be identified. Representatives from the sponsor site may be required to access patient notes for quality assurance purposes and will maintain confidentiality at all times. The site PI must maintain all study-related documents in strict confidence at each site.

Centres will be allocated a study code by the trial co-ordinator. Data identifying individual Trusts and sites will not be presented in any reports. If the PI at any site requests to see their own data, site data will be made available to that site in summary form, with only that specific site identified.

### Insurance and indemnity

NHS indemnity and professional indemnity will be provided by the sponsor to meet the potential legal liability of investigators/collaborators arising from harm to participants in the management and conduct of the research.

## Dissemination of study findings

Following data analysis, a detailed national report will be produced which summarises the aggregate data from all hospital sites that participated in the study. Data analysis is anticipated to commence 6 months after study enrolment and so the earliest anticipated date for publication is December 2017. In addition to study outcomes, the report will contain high-level aggregated data such as the number of Trusts using the care bundle; the extent to which the care bundle is being implemented by UK hospitals; the completeness and quality of data collected in line with the care bundle by Trusts; action plans for improvements in implementing the care bundle and public involvement in policy recommendations. The report will be reviewed by the all third-sector and professional stakeholders prior to release. Data relating to identifiable Trusts and Hospitals will not be published in any open reports or publications. If requested, Trusts may be provided with summaries of their own data in an appropriate format.

The University of Manchester’s research team will produce a lay summary of the main national report which highlights key high-level data from the study. The report will be presented in an infographic format which is accessible to all families and the wider public. The lay report will be accessible online and through social networking activities such as Twitter. The report will also be disseminated via our Third-Sector Stakeholders who are already fully engaged with the work of the care bundle.

Results of the study will be submitted for publication in a peer review journal following completion of the study. Manuscript(s) will be prepared by the study team and authorship will be mutually agreed prior to manuscript preparation. Neutral or negative results will not delay publication of findings. A lay summary of results will be made available to all participants at the end of the trial.

During the study, press releases regarding the study may be disseminated. All press releases will need prior authorisation from the sponsor and NHS England. No third party will be permitted to submit publicity material without prior approval from the sponsor. All conference presentations and abstracts relating to the study must be approved by the sponsor prior to submission to the event organiser or editors. All publications will acknowledge the support of NHS England in funding the study and the University of Manchester for sponsoring the study. All study sites and their participants must be acknowledged.

## Discussion

Stillbirth is a tragic and prevalent outcome of pregnancy associated with both psychological and physical morbidity and social cost to the affected families and broader community. The Saving Babies Lives care bundle has been developed from existing evidence-based guidance with the aim of reducing stillbirth in the UK. However, there is a paucity of high-grade evidence to inform stillbirth reduction programmes in high-income countries. In part, this is because interventional studies are prohibitively large. The authors recognise that the study design is pragmatic, being necessarily limited by the availability of data and the limited timescales and funding. In particular there was no opportunity to prospectively gather pre-intervention data or design a phased implementation such as a stepped-wedge trial. This study aims to make use of the available data to provide useful practice-based evidence to determine whether the Saving Babies Lives programme has a positive impact on process outcomes and whether this translates into improved clinical outcomes. This study will also consider the wider impact of implementing the care bundle by reporting on perceptions of maternity care from service-users and professionals, this will be valuable when considering the implementation of future large-scale implementation projects in maternity care. The evaluation will give information about the impact on resource use and the economic cost of delivering maternity care. It is anticipated that this information will enable clinicians, commissioners and policy-makers to assess whether the care bundle is achieving its aims and to refine the tool where necessary.

## Abbreviations

AGA: Appropriately Grown for Gestational Age
AGREE: Appraisal of Guidelines for Research and Evaluation
CO: Carbon Monoxide
CRN: Clinical Research Network
CTG: Cardiotocography
DCP: Data Collection Proforma
EBC: Each Baby Counts
EFW: Estimated Fetal Weight
FGR: Fetal Growth Restriction
FH: Fetal Heart
GAP: Growth Assessment Programme
GEE: General Estimating Equation
GLMM: Generalised Linear Mixed Model
GROW: Gestation Related Optimal Weight
HRA: Health Research Authority
ITS: Interrupted Time Series
MBRRACE: Mothers and Babies: Reducing Risk through Audit and Confidential Enquiries
MIS: Maternity Information System
MTP: Maternity Transformation Programme
NHS: National Health Service
NICU: Neonatal Intensive Care Unit
NIHR: National Institute for Health Research
ONS: Office for National Statistics
PI: Principal Investigator
REC: Research Ethics Council
RFM: Reduced Fetal Movements
SBL: Saving Babies Lives
SCN: Strategic Clinical Network
SFH: Symphysis Fundal Height
SGA: Small for Gestational Age
SPiRE: Saving Babies Lives’ Project Impact and Results Evaluation
SSC: Study Stakeholder Committee
TOR: Terms of Reference
